# Optimized Phase-Generated Carrier Demodulation Algorithm for Membrane-Free Fabry-Pérot Acoustic Sensor with High Sensitivity

**DOI:** 10.3390/mi16020196

**Published:** 2025-02-08

**Authors:** Yang Yang, Xinyu Zhao, Yongqiu Zheng, Juan Cui, Dongqing Zhao, Zhixuan Zheng, Yan Cao, Chenyang Xue

**Affiliations:** The Key Laboratory of Instrumentation Science and Dynamic Measurement Ministry of Education, North University of China, Taiyuan 030051, China; yangyanghcs@163.com (Y.Y.); zhaoxinyu_0803@163.com (X.Z.); cuijuan@nuc.edu.cn (J.C.); 20000127@nuc.edu.cn (D.Z.); zhengzhixuan7@163.com (Z.Z.); caoyan113334@163.com (Y.C.); xuechenyang@nuc.edu.cn (C.X.)

**Keywords:** acoustic demodulation system, Fabry–Pérot sensor, high sound pressure, phase-generated carrier algorithm

## Abstract

Demodulation of fiber optic Fabry–Pérot (F-P) acoustic sensors with high sensitivity and a large dynamic range continues to pose significant challenges. In this paper, we propose an advanced phase-generated carrier (PGC) demodulation algorithm, applied innovatively to membrane-free F-P acoustic sensors operating under high sound pressure. The algorithm optimizes acoustic demodulation results by adjusting the mixing phase delay, achieving the best signal to noise and distortion ratio (SINAD) and total harmonic distortion (THD) (<1%). Additionally, by introducing the cosine component of the acoustic signal obtained directly after filtering the interference signal, into the demodulation algorithm process, the sensitivity of the sensor at high sound pressure is significantly improved. The experimental results show that the ameliorated algorithm obtains a demodulation sensitivity of 34.95 μrad/Pa and a THD of 0.87%, both of which are superior to traditional PGC demodulation algorithms under the same experimental conditions. At the same time, the minimum detectable sound pressure of $129.73\;{\mathrm{mPa/Hz}}^{1/2}$ was obtained, and the sound pressure tested in the experiment at a frequency of 1 kHz was as high as 3169.78 Pa (164 dB). With the proposed algorithm, the flatness of the frequency response is ±0.82 dB from 100 Hz to 33 kHz, and a dynamic range of up to 102.6 dB was obtained, making it relevant in the field of aerospace acoustic measurements.

## 1. Introduction

Fiber optic Fabry–Pérot (F-P) acoustic sensors are widely used in industrial inspection [1], geological monitoring [2], and aerospace detections [3] due to their compact size, large dynamic range, and resistance to electromagnetic interference [4,5]. Over the past few decades, various structural designs of F-P sensors have been developed for acoustic detection applications [6]. Among these, an all solid-state, membrane-free F-P acoustic sensor has proven to be highly suitable for high sound pressure environments [7,8,9]. However, detection in high sound pressure environments also requires a large dynamic range and high sensitivity of the sensor. Since the performance of fiber optic acoustic sensors is directly influenced by demodulation methods, it is essential to develop a demodulation method that not only addresses the challenge of dynamic range in acoustic response but also ensures optimal frequency response and sensitivity.

Researchers have proposed various demodulation methods for F-P acoustic sensors, such as intensity demodulation [10], white light interferometry (WLI) demodulation [11], and phase demodulation [12]. The most commonly used of these is the intensity demodulation method [13,14]. Since the quadrature operating point (Q-point) of the interference spectrum is easily influenced by external environmental factors, Zhang et al. implemented a self-stabilizing demodulation system based on an operating point for F-P acoustic sensors [15]. However, the dynamic range of this system was constrained by the linear region, with a maximum detectable acoustic pressure limited to 136.9 dB, and the demodulation sensitivity was also affected by the slope at the Q-point on the scanned spectrum. Chen et al. employed phase modulation spectrum technology to demodulate the membrane-free acoustic sensor [7]. The theoretical maximum detectable sound pressure of this method was only 346.8 Pa, which was equivalent to a sound pressure level of 144.78 dB. Nonetheless, this demodulation method was exclusively suitable for high quality factor F-P sensors and is not appropriate for demodulating low-finesse F-P cavities. The WLI demodulation algorithm is also a widely used demodulation method for interferometric sensors [16,17]. This method typically required the use of a spectrometer to obtain the interference spectrum, which means the signal demodulation frequency was limited by the speed of the spectrometer, rendering it unsuitable for the demodulation of high-frequency acoustic signals. Zhang et al. proposed a four-wavelength quadrature phase demodulation technique for F-P sensors and dynamic signals [18]. Despite its excellent frequency response, this method was only effective for demodulating short-cavity F-P sensors.

The phase-generated carrier (PGC) demodulation method is a form of the phase demodulation technique characterized by a large dynamic range and high precision. This method employs high-frequency modulation by transferring acoustic signals to the sidebands of the carrier signal. The cosine and sine terms can be extracted by mixing the interferometric signals from the sensor with oscillating signals of appropriate frequency and phase [19]. The PGC demodulation method is suitable for membrane-free F-P acoustic sensors, as they possess relatively long cavities and low reflectivity for membrane-free sensors [19,20,21]. Traditional PGC demodulation methods, such as PGC-Arctan [22], PGC-DCM [23], and PGC-SDD [24], were affected by carrier phase delay, which introduces nonlinear factors that impact the total harmonic distortion (THD) of the demodulation results. Yan et al. proposed a precision PGC demodulation technique for a homodyne interferometer modulated with a combined sinusoidal and triangular signal [25]. This method mitigated the effects of carrier phase delay by using ellipse fitting. Nevertheless, the extensive computation required by the algorithm restricted it to be only used for low-frequency displacement signals rather than high-frequency acoustic signals. Yuan et al. developed a self-referenced PGC algorithm [26]. This algorithm reduced the effects of carrier phase delay, achieving good vibration demodulation results. However, when applied to demodulating membrane-free acoustic sensors, its demodulation sensitivity is relatively low.

In this paper, we introduce an improved PGC algorithm and develop a demodulation system for membrane-free F-P acoustic sensors. The algorithm compensates for the carrier phase delay and the mixing phase delay so that the demodulation result is at the optimal signal to noise and distortion ratio (SINAD) and THD positions, and then demodulates the acoustic signal by introducing the cosine component of the acoustic signal obtained directly after filtering the interference signal for demodulation. Furthermore, a demodulation system is built on the basis of this algorithm. The experiments are performed under the conditions established by a high sound pressure calibrator. The experimental results show that, based on the proposed PGC algorithm, a demodulation sensitivity of 34.95 μrad/Pa and a THD of 0.87% is realized, which are superior to conventional PGC-Arctan, PGC-SDD, and SR-PGC methods. Meanwhile, the dynamic range of this system is up to 102.6 dB. The flatness is ±0.82 dB in the frequency range from 100 Hz to 33 kHz. These results demonstrate that the improved PGC algorithm and demodulation system has an excellent sensitivity and frequency response, and a large dynamic range. With its high sensitivity and large dynamic range, the proposed demodulation method displays significant application potential in acoustic measurements in high sound pressure environments, particularly in fields like aerospace and the smelting industries.

## 2. Detection and Demodulation Algorithm Principles

**Figure 1:**
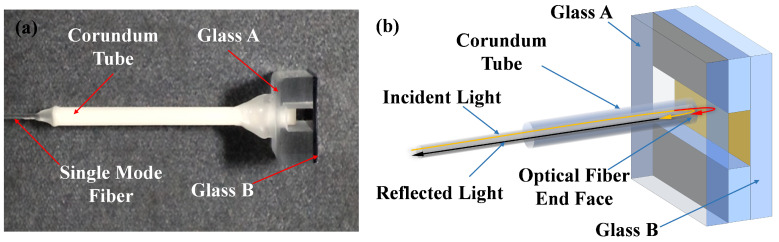
(**a**) An image of the membrane-free F-P acoustic sensor. (**b**) Interference principle for the sensor.

A low-finesse, membrane-free optical fiber F-P acoustic sensor is used for the experiments in this paper, as depicted in Figure 1a. The sensor has a cavity length of approximately 1093.8 μm, and mainly consists of an open cavity and a single-mode optical fiber, as shown in Figure 1b. The cavity is formed by two glass pieces, fixed together by spacers that are insensitive to temperature and other external environmental factors. The glass A, glass B, and spacers are made of ULE (ultra-Low expansion) glass, which consists of silicon dioxide (${\mathrm{SiO}}_{2}$) and titanium dioxide (${\mathrm{TiO}}_{2}$), and has a coefficient of thermal expansion that is close to zero—within the range of 0 to 50 °C. The glass B was given a surface treatment. This property allows it to maintain extremely high dimensional stability during temperature fluctuations. In addition, ULE glass has low thermal conductivity and excellent mechanical strength, making it suitable for use in high-precision optical components. The cavity length therefore hardly changes during the full duration of the 20 °C experiment. The single-mode fiber is threaded through a hole reserved in the middle of glass A, and its end face forms an F-P interferometric structure with the inner surface of glass B. This cavity is used to sensitize refractive index changes. A monochromatic laser is injected into a single-mode fiber and reflected at the fiber end face and glass B, respectively. Since the reflectivity of glass B is relatively low, the interference in this F-P cavity can be approximated as two-beam interference.

As the sound wave is transmitted into the air cavity of the acoustic sensor, the density of the air in the cavity is changed. This slight change in the air density affects the speed of laser transmission in the cavity, thus introducing an optical path difference between the two interfering laser beams. The phase difference caused by this optical path change in the acoustic sensor can be expressed as follows:

(1) $$\Delta \varphi =\frac{4\pi \cdot \Delta n\cdot L}{\lambda }$$

where Δn denotes the change of refractive index of air, *L* represents the cavity length of the sensor, and λ is the wavelength of the laser used. Since the cavity is a rigid structure, its cavity length remains constant as the acoustic signal is transmitted into the cavity. The only change occurs in the refractive index of air in the cavity, and eventually the interference spectrum changes. According to previous research [27], the relationship between the change in sound pressure and the change in the refractive index of air can be obtained by

(2) $$\Delta n_{ph}=\left[\frac{273.15}{101325}\times \frac{\Delta p}{T}\times \left(287.6155+\frac{1.62887}{\lambda^{2}}+\frac{0.01360}{\lambda^{4}}\right)\right]\times 10^{-6}$$

where, Δp indicates the change of sound pressure, *T* denotes temperature, and λ is the wavelength of the laser. As Equation (2) shows, it is evident that the change in the refractive index of air is linearly related to the change in sound pressure. At a temperature of 20 °C, with the laser operating at a central wavelength of 1550 nm, the refractive index of air changes by $2.65\times 10^{-9}$, with changes in sound pressure of 1 Pa.

**Figure 2:**
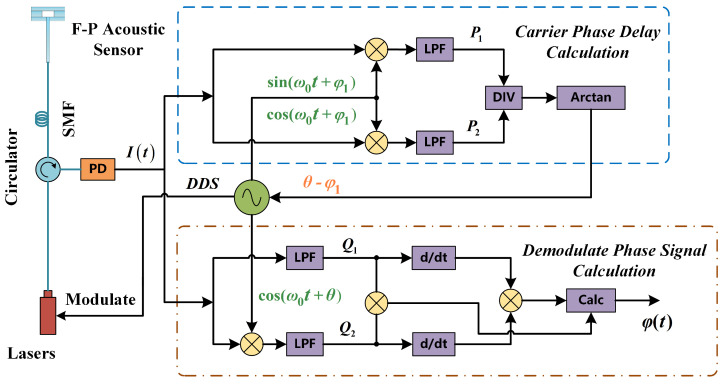
The principle of the proposed improved PGC demodulation algorithm.

Through detailed analysis of the interference principles of the acoustic sensor, it becomes clear that the minimal reflectivity of the sensor produces an interference pattern akin to that of two-beam interference. Hence, we have enhanced the traditional PGC algorithm, as depicted in Figure 2. Before being injected into the F-P acoustic sensor, the monochromatic laser is modulated into a frequency that far exceeds that of the acoustic signal. Subsequently, the interfered optical signal is converted into an electrical signal by the photodetector (PD). The electrical signal $I\left(t\right)$, resembling dual-beam interference, can be represented as follows:

(3) $$I\left(t\right)=A+B\cos\left(C\cos\left(\omega_{0}t+\theta \right)+\varphi \left(t\right)\right)$$

where *A* and *B* are constants related to the interfering light intensity, *C* respects the modulation depth of the laser, $\omega_{0}$ is the modulation frequency which must be far higher than the frequency of the acoustic signal, and θ is the carrier phase delay, which mainly originates from the current modulation of the laser. Rigorously, $\varphi \left(t\right)$ contains two components, one from environmental factors such as temperature (quasi-DC component) $\phi_{0}$ and one from changes in sound pressure (AC component) $\Delta \phi \left(t\right)$, i.e., $\varphi \left(t\right)=\Delta \phi \left(t\right)+\phi_{0}$. According Equation (3), we can obtain the following:

(4) $$\begin{array}{c} I\left(t\right)=A+B\left\{\left[J_{0}\left(C\right)+2\sum_{k=1}^{\infty }{\left(-1\right)}^{k}J_{2k}\left(C\right)\cos\left[2k\left(\omega_{0}t+\theta \right)\right]\right]\cdot \right.\\ \left.\cos\varphi \left(t\right)-\left[2\sum_{k=0}^{\infty }{\left(-1\right)}^{k}J_{2k+1}\left(C\right)\cos\left[\left(2k+1\right)\left(\omega_{0}t+\theta \right)\right]\right]\sin\varphi \left(t\right)\right\} \end{array}$$

where $J_{n}\left(C\right)$ is the $n^{th}$-order Bessel function. In the conventional PGC demodulation algorithm, the interfering signal introduces two phase delays when mixed with the carrier signal. One of these is the carrier phase delay θ mentioned above, which usually originates from the modulation output delay of the laser. The other is the phase difference $\varphi_{1}$ between the interfering signal and the carrier signal when mixing. Since these delays can affect the THD and SINAD of the acoustic demodulation results, it is necessary to compensate for both delays.

Two signals with the same frequency and a phase difference of π/2 are generated by the direct digital synthesizer (DDS) module as the carrier signals. These two signals can be expressed as follows:

(5) $$I_{a}=\sin\left(\omega_{0}t+\varphi_{1}\right)$$

(6) $$I_{b}=\cos\left(\omega_{0}t+\varphi_{1}\right)$$

After mixing with the interference signal and low-pass filtering, respectively,

(7) $$P_{1}=LPF\left[I\left(t\right)\cdot I_{a}\right]=-BJ_{1}\left(C\right)\sin\left(\theta -\varphi_{1}\right)\sin\varphi \left(t\right)$$

(8) $$P_{2}=LPF\left[I\left(t\right)\cdot I_{b}\right]=-BJ_{1}\left(C\right)\cos\left(\theta -\varphi_{1}\right)\sin\varphi \left(t\right)$$

where $LPF\left[*\right]$ refers to low-pass filtering. The above Equations (7) and (8) can be divided and arctangent to obtain the total phase delay value:

(9) $$\mathrm{Arctan}\left[\frac{P_{1}}{P_{2}}\right]=\theta -\varphi_{1}$$

**Figure 3:**
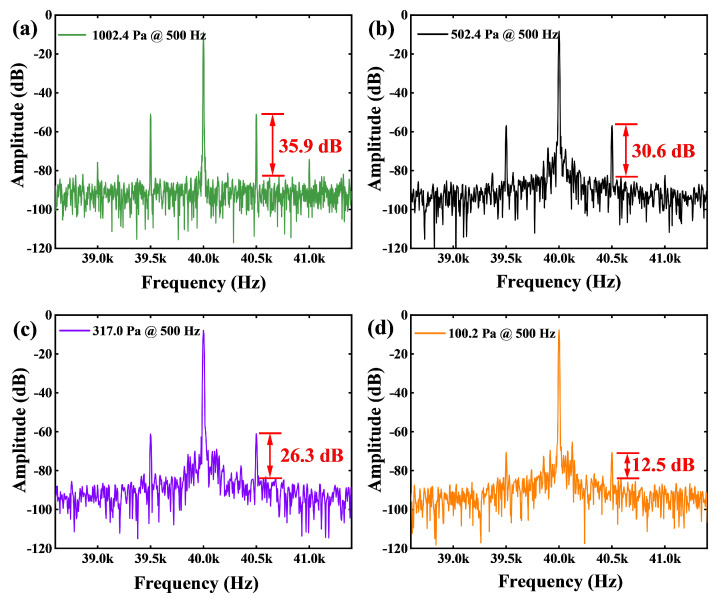
The amplitude of the acoustic signal components in the second harmonic sidebands of the interference signal under different sound pressures. (**a**) The sound pressure is 1002.4 Pa. (**b**) The sound pressure is 502.4 Pa. (**c**) The sound pressure is 317.0 Pa. (**d**) The sound pressure is 100.2 Pa.

Before processing the interference signal $I\left(t\right)$, we analyze the interference spectra at a 20 kHz laser modulation frequency when the acoustic signal frequency is fixed at 500 Hz with sound pressures of 1002.4 Pa, 502.4 Pa, 317.0 Pa, and 100.2 Pa applied, respectively. Figure 3 illustrates the amplitude of the acoustic signal components in the second harmonic sideband of the interference spectrum under different sound pressures. It can be observed that as the sound pressure decreases, the amplitude of the acoustic signal components also diminishes. When the sound pressure drops below a certain threshold, effective acoustic signals can no longer be extracted using the traditional mixing method.

Hence, to achieve better demodulation sensitivity and dynamic range, the interfering signal $I\left(t\right)$ is split into two paths after passing through the PD, as shown in Figure 2. One path is low-pass filtered directly, and the other path is low-pass filtered after mixing, first with harmonic components of the carrier signal, followed by the compensation for the phase using the previously calculated delay value $\theta -\varphi_{1}$. Accordingly, a pair of orthogonal signals is obtained through the above two paths:

(10) $$Q_{1}=LPF\left[I\left(t\right)\right]=BJ_{0}\left(C\right)\cos\varphi \left(t\right)$$

(11) $$Q_{2}=LPF\left[I\left(t\right)\cdot \cos\left(\omega_{0}t+\theta \right)\right]=-BJ_{1}\left(C\right)\sin\varphi \left(t\right)$$

Next, differentiating Equation (10) and Equation (11), respectively,

(12) $${Q_{1}}^{\prime }=-BJ_{0}\left(C\right)\varphi^{\prime }\left(t\right)\sin\varphi \left(t\right)$$

(13) $${Q_{2}}^{\prime }=-BJ_{1}\left(C\right)\varphi^{\prime }\left(t\right)\cos\varphi \left(t\right)$$

According to Equations (10)–(13), we can derive the following:

(14) $$\frac{{Q_{1}}^{\prime }\cdot {Q_{2}}^{\prime }}{Q_{1}\cdot Q_{2}}=-{\left[\varphi^{\prime }\left(t\right)\right]}^{2}$$

The above signals can be demodulated to $\varphi \left(t\right)$ by inverting, rooting, symbol recovery, and integration. It can be seen that among the differentiation of $\varphi \left(t\right)$, the quasi-DC component $\phi_{0}$ differentiation results are close to zero. After integration, we used an appropriate filter that retained only the high-frequency dynamics of the acoustic signal, so that during the experiments at an environmental temperature of 20 °C, the effect of temperature on the demodulation results was not significant. We use the variation of $\Delta \phi \left(t\right)$ to characterize the performance of the acoustic sensor. It can also be seen that the demodulated results do not contain nonlinear components, which theoretically indicates that the algorithm is helpful in improving the THD of the demodulated results.

(15) $$S_{out}=\Delta \phi \left(t\right)$$

## 3. Construction of the Demodulation System

**Figure 4:**
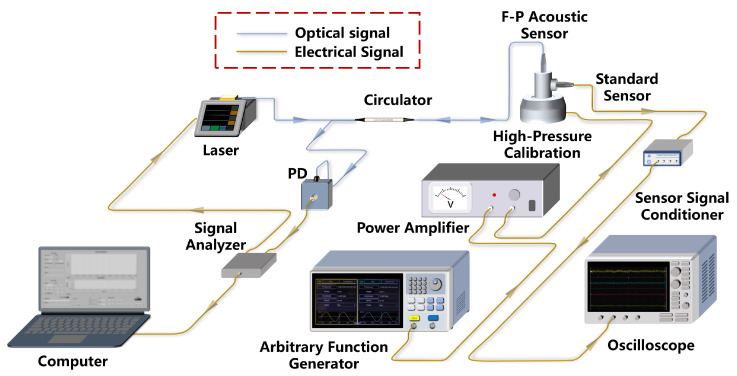
Schematic of membrane-free fiber optic F-P acoustic sensor demodulation system.

In order to verify the demodulation performance of this demodulation algorithm for the membrane-free F-P acoustic sensor under high sound pressure, we construct a demodulation system, as shown in Figure 4. The system employs a DBF laser (DBF1550P, Thorlabs, Newton, NJ, USA) with a central wavelength of 1550 nm, a tunable range of 4.5 nm, and a linewidth of 150 kHz. The laser is driven by a compact laser controller (CLD1015, Thorlabs), which has a maximum current modulation frequency of 250 kHz and therefore a wide range of sound bands that can be demodulated. This experimental system uses a standard electroacoustic sensor (378C01, PCB, Depew, NY, USA) for the calibration of sound pressure levels. The sound pressure information of the standard electroacoustic sensor is output as an electrical signal to an oscilloscope for display using the sensor signal conditioner (482C, PCB). The sound source uses a high sound pressure calibrator. A standard sine signal generated by the Arbitrary Function Generator (31102, Tektronix, Beaverton, OR, USA) is amplified by a power amplifier to drive the calibrator. The maximum sound pressure level that this high-pressure calibration can achieve is 164 dB. A PD (2117, NEW Focus, San Jose, CA, USA) is used to convert the interfering light intensity of the F-P sensor into voltage signals. The signal analyzer captures the voltage signal and simultaneously generates the modulation signal for the laser. Since the laser allows modulation frequencies up to 250 kHz, we opted for internal modulation to reduce system complexity.

When the acoustic signal enters the F-P cavity, the modulated laser beam passes through the optical circulator and enters the F-P acoustic sensor simultaneously, and the resulting phase shift is superimposed onto the carrier signal. The reflected light passes through the optical circulator into the PD, and the PD outputs an electrical signal representing the interfering light intensity. A signal analyzer based on a field-programmable gate array platform is used as the demodulation hardware, and we integrate part of the PGC algorithm into this platform. The processed signals are then transferred to a computer for subsequent algorithm processing, and the demodulation results are finally displayed to the computer.

It is clear that the implementation of the demodulation function of a constructed acoustic demodulation system primarily relies on the proposed PGC demodulation algorithm, which avoids the requirements for complex optical path designs. As a result, this demodulation system is exceptionally well suited for acoustic signal demodulation in harsh environments.

## 4. Experimental Results and Discussion

**Figure 5:**
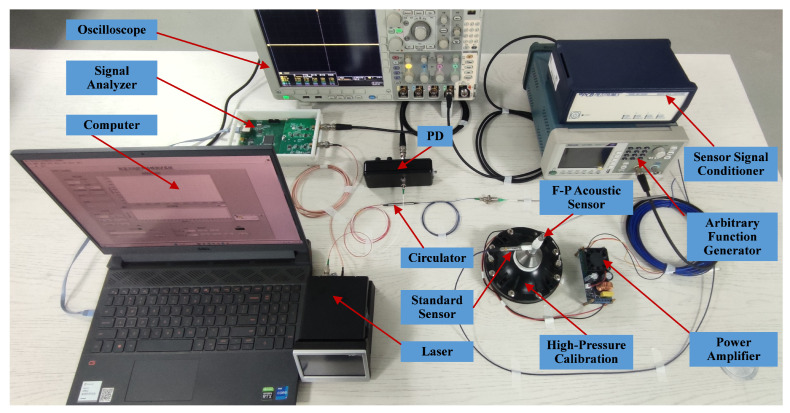
Construction of the acoustic sensing system.

**Figure 6:**
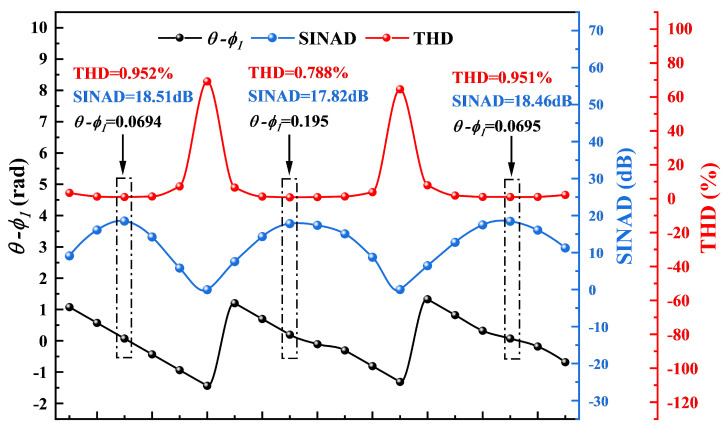
The impact on the THD and SINAD of the demodulated acoustic signal.

To validate the demodulation performance of the proposed PGC algorithm for membrane-free acoustic sensors, we construct experiments using the system depicted in Figure 5. A sinusoidal electrical signal generated by the signal analyzer is used to modulate the laser. As analyzed above, the traditional PGC algorithm introduces a carrier phase delay of θ and a mixing phase error of $\phi_{1}$. In order to assess the impact of the phase error caused by these two parts on the acoustic signal demodulation results, the impact of the total delay error on the demodulation results, including THD and SINAD, is analyzed by adjusting the phase of the mixing signal. This mixing signal is generated by the DDS module of the signal analyzer. Based on the experimental results, the variation of $\theta -\phi_{1}$ is calculated by applying Equation (9). Then, the SINAD and THD of the demodulated acoustic signal are analyzed, as illustrated in Figure 6. Phase shifts in the mixing signals lead to changes in the $\theta -\phi_{1}$ within the range of ±π/2. When $\theta -\phi_{1}$ approaches ±π/2, the SINAD of the demodulated acoustic signal reaches its minimum, while THD increases and signal distortion becomes severe. With $\theta -\phi_{1}$ approaching zero, the acoustic signal achieves minimal THD and maximal SINAD, and demonstrates that the change is cyclical. Therefore, we adjust the phase of the mixing signal to optimize THD and SINAD in the acoustic demodulation results before proceeding with the subsequent experiments.

**Figure 7:**
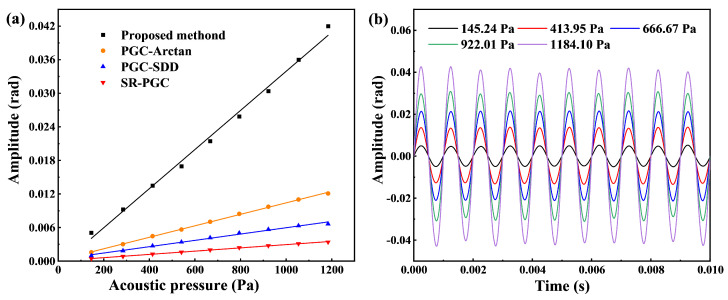
(**a**) The linear fit of phase versus sound pressure for different PGC demodulation algorithms. (**b**) Time response of the F-P acoustic sensor under different sound pressures at 1 kHz based on the proposed PGC algorithm.

Subsequently, we conduct sensitivity experiments on the membrane-free F-P acoustic sensor using the proposed PGC algorithm. Sensitivity is a key measure of the responsiveness of the entire demodulation system to sound pressure. Initially, a standard sinusoidal signal with a fixed frequency of 1 kHz is generated by the AFG. After passing through a power amplifier, this signal was utilized to drive the high sound pressure calibrator. By adjusting the amplitude of the sinusoidal signal, the output sound pressure level of the high sound pressure calibrator can be effectively controlled. On the other hand, a calibrated standard electroacoustic sensor is utilized to obtain accurate sound pressure values. Figure 7a shows the relationship between the amplitude of the acoustic signal and the sound pressure. It can be observed that the slope of the linear fit curve of the improved PGC algorithm is $3.495\times 10^{-5}$, i.e., the demodulation sensitivity of the algorithm is 34.95 μrad/Pa. The linear fit of $R^{2}$ is 0.9944, indicating excellent linearity of the proposed method. In order to compare the demodulation sensitivity of the proposed improved PGC algorithm with that of traditional PGC algorithms, interference signals at the same modulation depth are demodulated using different algorithms and the results are analyzed, as shown in Figure 7b. The demodulation sensitivities of the conventional PGC-Arctan and PGC-SDD algorithms are 10.26 μrad/Pa and 5.64 μrad/Pa, respectively, while the demodulation sensitivity of the SR-PGC algorithm is only 2.91 μrad/Pa. These results demonstrate that the demodulation sensitivity of the proposed improved PGC algorithm is significantly better than that of the conventional PGC algorithms. Figure 7b illustrates the variation in the demodulated signal amplitude with time for the improved PGC algorithm at sound pressures of 145.24 Pa, 413.95 Pa, 666.67 Pa, 922.01 Pa, and 1184.10 Pa, respectively.

**Figure 8:**
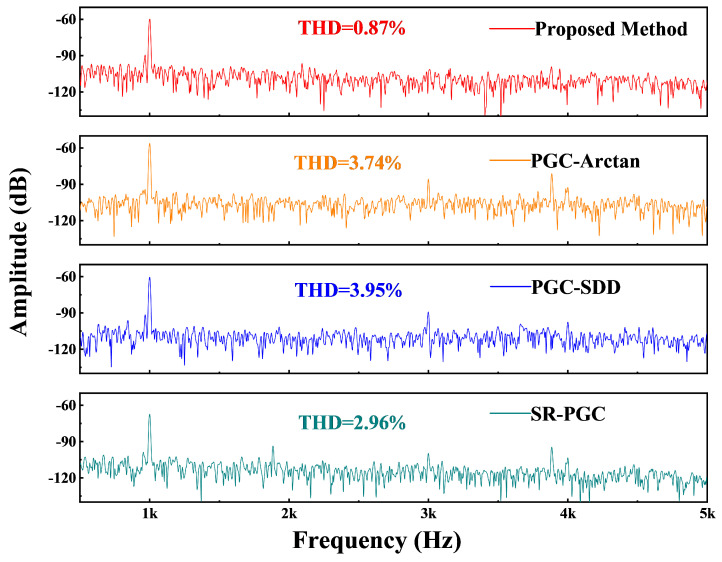
THD of acoustic demodulation results under different PGC algorithms.

The previously derived Equation (15) indicates that there is no nonlinear error in the proposed method. However, nonlinear distortion features are present in some traditional PGC algorithms like PGC-Arctan. Consequently, we proceed to evaluate the nonlinear distortion characteristics of the demodulated signal generated by the proposed algorithm. As shown in Figure 8, the proposed PGC algorithm achieves a 0.87% THD at 1 kHz and 145.24 Pa. Meanwhile, we compare the THD of the acoustic signal across different PGC demodulation algorithms under the same acoustic conditions. Experimental results indicate that the same raw interfering signals demodulated using the PGC-Arctan, PGC-SDD, and SR-PGC algorithms resulted in THD values of 3.74%, 3.95%, and 2.96%, respectively. All three algorithms exhibit significant harmonic signals at 3 kHz and 4 kHz. Moreover, the amplitude of the signals demodulated by these three methods under the same sound pressure is noticeably lower than that of the proposed method, which is a key factor contributing to the higher harmonic distortion in these methods. Overall, the proposed method demonstrates a slightly lower THD.

We then analyzed the resource usage of the different algorithms. We have divided the algorithm into two parts; PGC-Arctan, PGC-DCM, and both SR-PGC and the proposed method require operations such as mixing and filtering. In the first part, we run the mixing and filtering algorithms on the ZYNQ platform, which reduces the time used for computation by taking advantage of its hardware multipliers as well as its high-speed parallelism. Since the various algorithms are similar in mixing and filtering, in terms of computational resources, each algorithm is basically the same in terms of resource consumption and computation time, with a total delay time of about 875 ns. The other part is due to the fact that the subsequent processing of various algorithms is not the same; we transfer the mixing and filtering data to the software built by labview (version 2020) for processing. In terms of algorithmic complexity, the SR-PGC algorithm is more complex, takes the longest time to compute in the computer, and consumes more resources. The proposed method and the PGC-DCM algorithm involve a comparable amount of computation, and the one that consumes the least amount of resources is the PGC-Arctan algorithm due to its more concise algorithm. In the future research, we can consider porting all the computational processes to the ZYNQ platform, and utilize the ZYNQ platform to hardware accelerate the proposed demodulation algorithm in order to improve the performance of demodulation.

(16) $$D<\frac{\omega_{0}}{\left(2+b\right)\omega_{s}}-1$$

**Figure 9:**
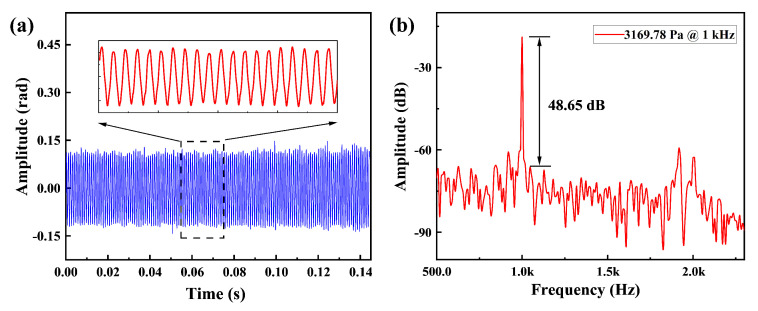
Time-domain waveform (**a**) and frequency-domain response (**b**) of the demodulated phase under an acoustic pressure of 3169.78 Pa at 1 kHz.

To test the dynamic range of this demodulation system, we adjust the output voltage amplitude of the AFG, and the high sound pressure calibrator reached a maximum sound pressure of 3169.78 Pa. Figure 9a presents the time-domain variation in the acoustic signal at the maximum sound pressure, and Figure 9b illustrates the spectral analysis of the demodulated acoustic signal. The results demonstrate that the proposed PGC demodulation method delivers stable demodulation results under high sound pressure conditions. Due to reaching the maximum sound pressure level of the high-pressure calibrator, we do not conduct tests at higher sound pressure levels. The maximum amplitude of the signal that can be demodulated by this algorithm is determined by the signal frequency $\omega_{s}$, the modulation frequency $\omega_{0}$, and the octave *b* of the filter, according to Equation (16). Where *D* is the amplitude of $\varphi \left(t\right)$. With a signal frequency of 33 kHz, a laser modulation frequency of 200 kHz, and an eighth-order low-pass filter, the theoretical maximum detectable sound pressure is 17,384 Pa (178.8 dB), based on the sensitivity of the system described above. It can be seen that the system has the potential to handle tests at higher sound pressure levels. As can be seen from Equation (16), the ratio of the modulation frequency and the acoustic signal frequency determines the dynamic range of the system, and the width of the low-pass filter transition band will also have a limiting effect on the dynamic range of the system; the larger the width of the transition band, the smaller the dynamic range of the system. Due to the infinite series property of Bessel function, spectral aliasing occurs when the sampling frequency is not infinite, the cause of aliasing can be understood as the overlap of the signal to be measured with the adjacent level subcarrier sideband signal in the spectrum, and the ratio of the modulation frequency to the acoustic signal frequency and the signal amplitude *D* determines the degree of spectral aliasing. The experimental situation shows that the ratio of the modulation frequency to the frequency of the acoustic signal should be greater than six in order to ensure the dynamic range of the system, i.e., the maximum detectable acoustic pressure of 17,384 Pa.

We then conduct experiments to determine the minimum detectable sound pressure (MDP). As shown in Table 1, at an input sound pressure of 3.027 Pa, the signal to noise ratio (SNR) was 16.39 dB, and the resolution bandwidth was 12.5 Hz, resulting in an MDP of $129.73\;{\mathrm{mPa/Hz}}^{1/2}$. With reference to a sound pressure of 20 μ${\mathrm{Pa/Hz}}^{1/2}$ in air, the minimum detectable sound pressure level is 76.2 dB. This acoustic sensing system has a dynamic range of up to 102.6 dB. Under the same input sound pressure, the traditional PGC demodulation algorithms, including PGC-Arctan, PGC-DCM, and PGC-SDD, achieve SNR values of 3.79 dB, 3.85 dB, and 1.99 dB, respectively, as shown in Table 1. The corresponding calculated minimum detectable sound pressures were $553.42\;{\mathrm{mPa/Hz}}^{1/2}$, $549.61\;{\mathrm{mPa/Hz}}^{1/2}$, and $680.86\;{\mathrm{mPa/Hz}}^{1/2}$, respectively. In contrast, the SR-PGC algorithm did not show a significant acoustic response until the input sound pressure reached 12.33 Pa, with the calculated minimum detectable sound pressure being $3.02\;{\mathrm{Pa/Hz}}^{1/2}$. Through comparison, it can be observed that although the proposed algorithm does not achieve a very low MDP, which may be due to the excessive noise introduced by directly filtering the spectral signal, it still demonstrates a significant advantage in MDP compared to other traditional PGC algorithms.

**Figure 10:**
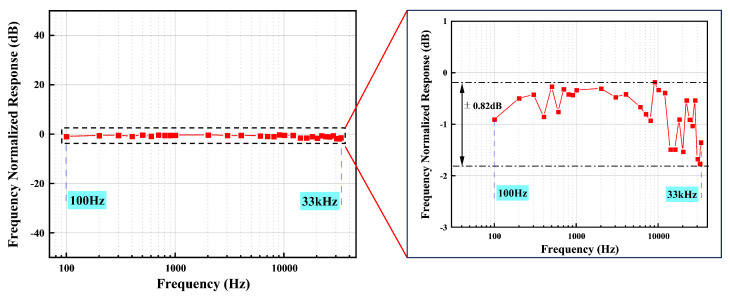
Frequency response and flatness of the demodulation system.

Frequency response is also an important metric in acoustic sensing systems, as it characterizes the range of frequencies to which the system is most sensitive. We test the frequency response of the demodulation system at modulation frequencies up to 200 kHz. Experimental results are depicted in Figure 10, showing that the improved PGC algorithm achieves a frequency response from 100 Hz to 33 kHz with a flatness of ±0.82 dB. In this system, as the modulation frequency increases, not only is there a delay in phase output, but the optical power also experiences varying degrees of attenuation. The amplitude of the optical power will directly affect the demodulation result of the system. This is one of the reasons why higher frequency signals cannot be used for modulation. However, the demodulation system constructed has a good frequency response while maintaining the sensitivity and THD of the demodulation results. In future work, by adjusting the modulation frequency or employing higher-frequency external modulation methods, it may be possible to extend the frequency response of the system.

## 5. Conclusions

In conclusion, we propose an improved PGC demodulation algorithm and build an acoustic sensing system with high sensitivity and a large dynamic range for the membrane-free F-P acoustic sensor. Theoretical analysis and experiments have both demonstrated that carrier phase delay and mixing phase delay affect the demodulation results. By optimizing the phase alignment of carrier phase delays and mixing phase delays, we achieve the best THD and SINAD in the demodulated acoustic signal. The demodulation algorithm significantly improves sensitivity to 34.95 μrad/Pa and reduces THD to 0.87% by introducing the first harmonic of the acoustic sensor interference signal, outperforming traditional methods such as PGC-Arctan, PGC-SDD, and SR-PGC. Stable demodulation results are also achieved under sound pressure levels as high as 3169.78 Pa, and up to 102.6 dB dynamic range is obtained. The frequency bandwidth is 100 Hz to 33 kHz, with a flatness of ±0.82 dB at 200 kHz laser modulation frequency. The experimental results demonstrate that the proposed demodulation system possesses exceptional demodulation sensitivity and dynamic range, enabling it to perform acoustic detection in harsh environments, such as aerospace applications.

## Figures and Tables

**Table 1:** Comparison of MDP in different PGC demodulation algorithms.

Demodulation Method	SNR	MDP	Ref.
PGC-Arctan	3.79 dB	$553.42\;{\mathrm{mPa/Hz}}^{1/2}$	[22]
PGC-SDD	1.99 dB	$680.86\;{\mathrm{mPa/Hz}}^{1/2}$	[24]
PGC-DCM	3.85 dB	$549.61\;{\mathrm{mPa/Hz}}^{1/2}$	[23]
SR-PGC	1.26 dB	$3.02\;{\mathrm{Pa/Hz}}^{1/2}$	[26]
Proposed Method	16.39 dB	$129.73\;{\mathrm{mPa/Hz}}^{1/2}$	This work

## Data Availability

The data presented in this study are available on request from the corresponding author.
